# Neonatal osteofibrous dysplasia: Synchronous tibial and fibular involvement is common

**DOI:** 10.1259/bjrcr.20210072

**Published:** 2022-01-24

**Authors:** Harsimran Laidlow-Singh, Pranai Buddhdev, Mark Latimer, Pearl Wou, Amaka C. Offiah

**Affiliations:** 1 Department of Radiology, Broomfield Hospital, Chelmsford, United Kingdom; 2 Department of Orthopaedic Surgery, Broomfield Hospital, Chelmsford, United Kingdom; 3 Department of Orthopaedic Surgery, Addenbrooke’s Hospital, Cambridge, United Kingdom; 4 Academic Unit of Child Health, University of Sheffield, Sheffield, United Kingdom

## Abstract

Osteofibrous dysplasia is a rare non-malignant fibro-osseous bone tumour, first described and characterised under this name by Campanacci in 1976. It is most commonly encountered in the tibia of children and young adults, but less frequently seen in the neonate with only few prior reports in the literature. We report a case of neonatal congenital osteofibrous dysplasia, presenting with unilateral limb deformity at birth. Radiographs demonstrated well-defined mixed lytic-sclerotic lesions, in a previously unreported distribution in this age-group, confined to the distal metadiaphysis of the affected tibia and fibula. Open surgery was performed for deformity correction, with tissue biopsy confirming the radiographically-suspected diagnosis. We present the up-to-date clinical, radiological and pathological findings in this case of a rare pathology with some novel features, within this age group, in disease distribution and consequent radiographic appearances. OFD should be considered in the differential of similar congenital deforming bone lesions of the lower limb. We also review the small number of previously published cases of congenital OFD in the neonate, noting in particular that the frequency of ipsilateral fibular involvement appears to be higher than that observed in older patients.

## Case report and imaging findings

**Figure 1.**
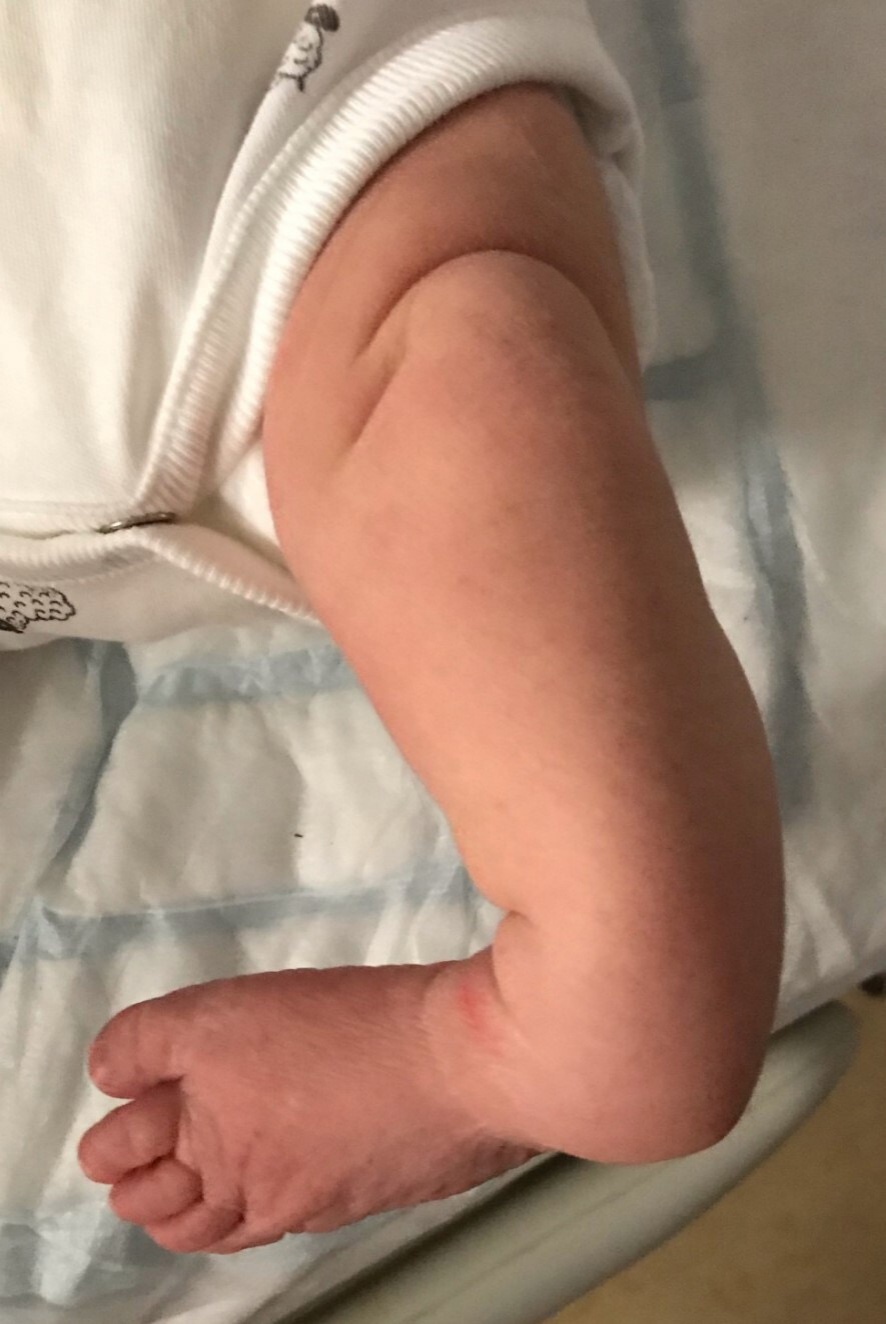
Clinical photograph demonstrating the left lower limb deformity noted at birth

**Figure 2.**
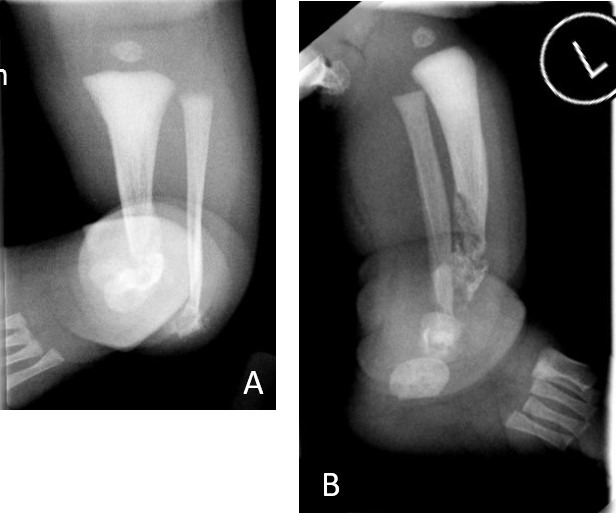
Anteroposterior (**A**) and lateral radiographs (**B**) of the left ankle at presentation (on day of birth) demonstrating mixed lytic-sclerotic lesions of the distal tibia and fibula with associated deformity

A female term neonate was noted at birth to have a deformity of her left lower limb/ankle with an anterolateral tibial bow associated with marked varus angulation and procurvatum (Figure 1). Delivery was by lower segment Caesarean section due to failure to progress at a gestational age of 40 + 4 weeks, and was otherwise uncomplicated. Routine antenatal ultrasound screening did not demonstrate any anomaly, although intrinsic lesions of bone are not typically identified antenetally. Clinical examination was otherwise normal, including no stigmata of neurofibromatosis. Radiographs of the affected extremity (Figure 2) confirmed anterolateral bowing of the tibia and fibula (which were hypoplastic when compared to the unaffected right leg) and focal osteolytic lesions of the distal tibial and fibular metadiaphyses, with extensive surrounding and internal patchy sclerosis. A skeletal survey did not demonstrate any further osseous lesions, and she was otherwise developmentally normal. Tertiary radiological opinion suggested the likely diagnosis to be osteofibrous dysplasia.

**Figure 3.**
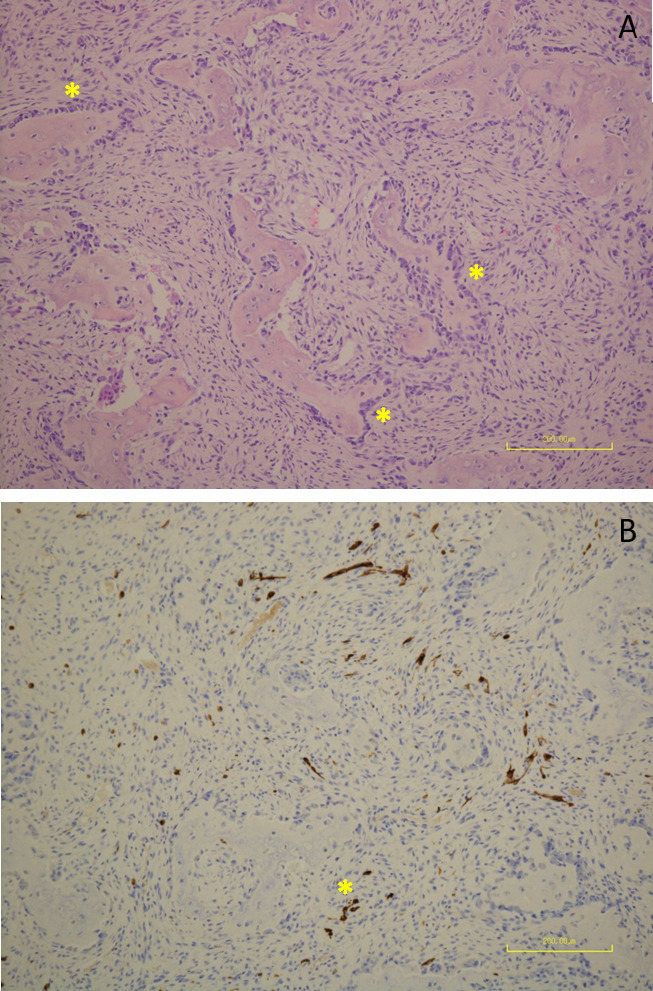
Photomicrographs, H&E stained section, x10 magnification. Demonstrating (**A**): tumour composed of irregular woven bone trabeculae with conspicuous osteoblastic rimming [*]. Intervening collagenous stroma with monotonous spindle cells. (**B**): Further section showing the stroma composed of bland spindle cells embedded in a collagenous matrix. Focal ossification is noted. Cytokeratin MNF116 stain showing single dispersed positive cells [*], confirming the diagnosis of congenital osteofibrous dysplasia

After multidisciplinary discussion at the local tertiary paediatric orthopaedic centre and at a national skeletal dysplasia meeting, surgery was performed at 3 months of age, for correction of the worsening clinical deformity by way of fibular and tibial osteotomy, temporary K-wire fixation and immobilisation in plaster. This afforded the opportunity for open biopsy, with histological analysis supporting the radiographic diagnosis, finding typical appearances of osteofibrous dysplasia^1–5^ including irregular woven bone trabeculae with prominent osteoblastic rimming, and bland spindle cells in a fibrous stroma, without features of malignancy or alternative pathology. Cytokeratin staining demonstrated only single dispersed positive cells (Figure 3A and B).

**Figure 4.**
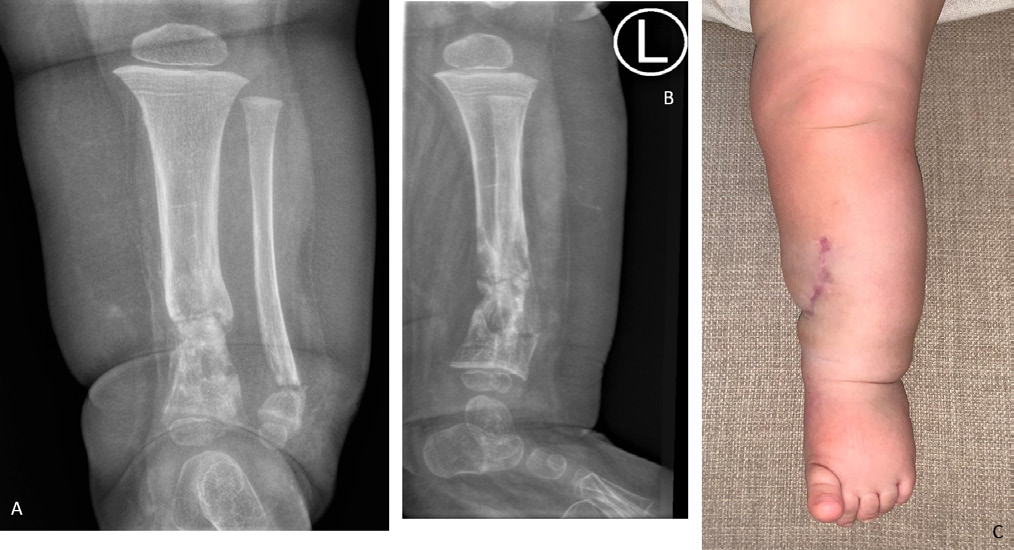
Anteroposterior (**A**) and lateral radiographs (**B**) of the affected limb (aged 14 months). There is post-surgical new bone formation with defined cortices of the distal tibia and fibula, and reactive sclerosis at the osteotomy sites. The focal lesion has a non-aggressive radiological appearance and the proximal diaphyses remain spared. (**C**): Clinical photograph demonstrating orthogonal alignment of the limb and minimal visual deformity as compared with Figure 1

The patient is followed-up locally and at a tertiary paediatric hospital. The most recent follow-up radiographs (Figure 4, at 14 months old) demonstrate a persistent mixed lytic-sclerotic lesion in the distal metadiaphyses of the tibia and fibula, with no obvious physeal or epiphyseal involvement, nor progression more proximally in the bone. There is a good level of deformity correction post-osteotomy, both radiologically (Figure 4A and B) and cosmetically (Figure 4C). The patient demonstrates preserved function and is ambulating independently with the aid of a clamshell orthosis, consistent with normal motor developmental milestones for age. There is good parental satisfaction with the current state and alignment of her limb, reporting improved comfort and mobility following surgery.

## Discussion and differential diagnosis

Congenital osteofibrous dysplasia presenting at birth is extremely rare in the reported literature (7 cases including ours) and remains unusual in its more typical patient population of older children. We summarise the lesion distribution, outcome after follow-up, and other clinical features of the prior six congenital neonatal cases in Table 1. In the majority of previously reported cases (5/6, 83%) either the proximal or entire tibia was affected,^2,3,6–8^ and in one the process was isolated to the distal fibula.^9^ Our case demonstrates that a synchronous distal tibial and distal fibular distribution is also compatible with the diagnosis, and is to our knowledge the first in the literature to demonstrate this pattern, in a neonate, with accompanying radiographs and clinico-pathological data.

**Table 1.** Summary of Previously Reported Cases of Congenital Osteofibrous Dysplasia

Author(s)	Publication and Date	Lesion Distribution	Both Tibia/Fibula Involved	Diagnostic Methods and Management	Stated Outcome
Hisaoka *et al*	Skeletal Radiology, 2004	Distal fibula	No	XR, MRI, en bloc resection and specimen histology	No complication at 1 year
Sarisozen *et al*	European Journal of Orthopaedic Surgery & Traumatology, 2005	Proximal tibial diametaphysis, later distal fibula	Yes	XR, CT, open biopsy. No resection.	No complication at 4 years
Teo *et al*	Skeletal Radiology, 2007	Entire tibia and distal fibula	Yes	XR, MRI, osteotomy and open biopsy. No resection.	No complication at 46 months
Zamzam	Saudi Medical Journal, 2008	Proximal tibial diaphysis and distal fibula	Yes	XR, MRI, NM scintigraphy, curettage with bone graft	No complication at 7 years
Jobke *et al*	Journal of Paediatric Haematology/Oncology, 2014	Proximal tibial diametaphysis	No	XR, open biopsy. No resection.	Lesion regression at 3.5 months, no complication at 1 year
Kim and Lee	Journal of the Korean Society of Radiology, 2015	Proximal tibial diametaphysis, later distal fibula	Yes	XR, MRI, open biopsy. No resection.	Not stated

We also note with interest that of the six previously reported neonatal cases (Table 1), ^2,3,6–9^ and the current patient, 71% (5/7) exhibit changes within both tibia and fibula either at presentation or after follow-up. This contrasts markedly to the much lower frequency of ipsilateral fibular involvement in published series of older children and young adults, of 12–20%.^1,5,10^ In those series the tibia, and often specifically its proximal part, is described as the typical location of osteofibrous dysplasia.^1,4,5,10^ In this particular regard, that which is uncommon in the adolescent appears the norm in the neonate and viceversa. It has been stated that OFD has “a predilection for the tibia in children”,^4^ to which we suggest the fibula should be added in neonatal cases.

Neither the presence of fibular involvement nor the distal distribution within the bones should, therefore, dissuade the radiologist from making the diagnosis of neonatal congenital osteofibrous dysplasia in an otherwise appropriate setting.

The radiologist’s role in neonatal osteofibrous dysplasia is likely to primarily lie in differential diagnosis. Some plausible differentials of lower limb bony deformity at birth are readily excluded on clinical or radiological grounds, for example trauma and congenital talipes equinovarus. Others can be differentiated by non-image based diagnostics, such as genetic testing in the case of neurofibromatosis Type 1, which would also be expected to have neurocutaneous stigmata in addition to deformity and a focal osseous lesion. When radiographs demonstrate typical osteofibrous dysplasia-like appearances of a mixed lytic and sclerotic bone lesion associated with deformity in the tibia of a neonate, with or without fibular involvement, there is a more limited differential. In particular, the radiographic distinction from adamantinoma and fibrous dysplasia may pose difficulty, and in an older child the former is a specific concern due to the potential for malignancy and hence requirement for more aggressive treatment.^4–6^ However, to the best of our knowledge there are no reported occurrences of congenital lesions diagnosed in the immediate neonatal period proven to represent either process, with a literature search demonstrating no published such cases.^11^ It remains plausible that these conditions could occur in the neonate but would be exceedingly rare and should only therefore be considered in the context of significant radiographic or histological evidence in their favour. It may be possible, as in this case, to suggest a diagnosis of neonatal congenital OFD radiographically with a high level of certainty, when the typical features are present.

We note that although cross-sectional imaging was commonly employed in the reported cases of neonatal congenital osteofibrous dysplasia, with 4/6 patients undergoing MRI and 1/6 CT (Table 1), no unique or specific features are described that contribute to the diagnostic process.^2,3,6–9^ When the radiographic appearances are convincing, therefore, the cost and complexity of cross-sectional imaging can be avoided. If confirmatory histology is desired, needle or open biopsy (as opposed to more aggressive surgery) is safe and does not risk failing to identify adamantinoma if it is accepted that it does not occur in the neonate. In 5/7 (71%) cases, including ours, no attempt at curative resection was made, with good long-term outcomes subsequently seen at follow-up intervals of 1–7 years (Table 1). These reassuring reports suggest, albeit from a small body of cases, that this histologically benign lesion has a natural history of resolution or stability and does not require radical management unless for secondary concerns, such as deformity correction as in our case.

## Learning points

1. Osteofibrous dysplasia, although rare, should be considered in the differential diagnosis of congenital deforming bone lesions of the lower limb of children and young adults. It has characteristic radiographic appearances, primarily comprising focal mixed lytic and sclerotic change centred on the cortex.
2. The disease process may affect any part of the tibia and, in neonates, commonly also the fibula. The involvement of the latter is less common in older children.
3. If confidently diagnosed, aggressive treatment including surgical resection is unnecessary, as neonatal OFD has a benign course.
